# Recent methodology advances in fluorescence molecular tomography

**DOI:** 10.1186/s42492-018-0001-6

**Published:** 2018-09-05

**Authors:** Yu An, Kun Wang, Jie Tian

**Affiliations:** Key Laboratory of Molecular Imaging, Institute of Automation, Chinese Academy of Sciences, Beijing, China

**Keywords:** Fluorescence molecular tomography, Image reconstruction, Photon propagation model, Forward problem, Inverse problem

## Abstract

Molecular imaging (MI) is a novel imaging discipline that has been continuously developed in recent years. It combines biochemistry, multimodal imaging, biomathematics, bioinformatics, cell & molecular physiology, biophysics, and pharmacology, and it provides a new technology platform for the early diagnosis and quantitative analysis of diseases, treatment monitoring and evaluation, and the development of comprehensive physiology. Fluorescence Molecular Tomography (FMT) is a type of optical imaging modality in MI that captures the three-dimensional distribution of fluorescence within a biological tissue generated by a specific molecule of fluorescent material within a biological tissue. Compared with other optical molecular imaging methods, FMT has the characteristics of high sensitivity, low cost, and safety and reliability. It has become the research frontier and research hotspot of optical molecular imaging technology. This paper took an overview of the recent methodology advances in FMT, mainly focused on the photon propagation model of FMT based on the radiative transfer equation (RTE), and the reconstruction problem solution consist of forward problem and inverse problem. We introduce the detailed technologies utilized in reconstruction of FMT. Finally, the challenges in FMT were discussed. This survey aims at summarizing current research hotspots in methodology of FMT, from which future research may benefit.

## Background

Optical molecular imaging (OMI) uses molecular probes to label target organisms. Under certain external conditions, the molecular probe releases fluorescent light in the visible or near-infrared spectrum, using high-sensitivity detection equipment for fluorescence. The signal is collected and the position and intensity of the fluorescent light source are displayed to obtain physiological activity information of the organism’s molecular cells. OMI has the advantages of high sensitivity, no radiation, low cost, dynamic observation, and intuitive imaging, and can achieve early detection of tumors. Therefore, OMI has been rapidly developed in recent years, and has been widely used in tumor detection, drug development, image-guide surgery and other fields [1–4].

Fluorescence molecular tomography (FMT) is a three-dimensional imaging method based on fluorescence molecular imaging (FMI), which is based on the distribution of fluorescence in biological tissues [2, 3, 5–10]. It develops from two-dimensional (2D) qualitative imaging to three-dimensional (3D) quantitative research, and further expands the integration of stimulated fluorescence in the diagnosis and treatment of cancer, preclinical and clinical applications such as pharmacokinetics. Compared to other OMI technologies, FMT has the characteristics of low cost, safety, reliability, high signal strength, and flexible and reliable imaging. It has developed rapidly in recent years and has become a research frontier and research hotspot for OMI technology [11–17].

## Methods

In FMT, first we need to get the precise photon propagation model for describing both excitation and emission light transmission in-vivo. When the imaging spatial data needed for FMT reconstruction is obtained, and the reconstruction of the structural data and optical data based on the biological model can be carried out [18]. In general, the image reconstruction process includes two steps: solving the forward problem and solving the inverse problem. The solution of the forward problem is to calculate the photon propagation model of the fluorescence transmitted in the imaging space to obtain the linear relationship between the fluorescence measurement data on the surface of the tissue and the fluorescence distribution inside the bio tissue. After the linear relationship is obtained by solving the photon transfer model, various methods are used to solve the linear model, and the distribution of fluorescence inside the imaging space is obtained, which is called inverse problem [6]. As a conclusion, there are mainly three components in FMT methodology, photon propagation model, forward problems solving and inverse problems solving. Next, we will briefly introduce the mainstream of current methods used in FMT reconstruction, and discusses the analysis of the characteristics of each method and its scope.

## Results and discussion

### Photon propagation model

The process of transmitting fluorescence from a light source to a biological body through a specific biological tissue is extremely complicated, and includes various physical processes such as scattering of light, inter-tissue reflection, refraction, diffusion and absorption. For FMT imaging, imaging is usually performed in the visible and near-infrared optical bands, and the scattering and absorption effects of this band of light inside the biological tissues are the main forms of our study. Therefore, the FMT photon propagation model can be simplified to a photon stochastic propagation model that contains only the scattering and absorption effects without considering the reflection and refraction of different tissues. Today’s mainstream mathematical theories to solve these problems include analytical theory and transport theory. The analytical theory is based on the Maxwell wave equation and takes into account the multiplicative scattering, wave interference and diffraction, and tissue absorption effects of photons, introduces the related physical processes into the wave equation, and through rigorous analytical derivation, the micro integral equation. However, in the actual FMT experiment, the calculus equations containing these complex effects are difficult to solve due to the large number of optical parameters to be obtained, and it is impossible to get exact solutions of these optical parameters at the same time. It can be utilized only in the combination of some a priori knowledge of the circumstances, while narrow the scope of the solution of these optical parameters. Transport theory is based on Boltzmann’s Radiative Transfer Equation (RTE) [19], which is equivalent to photon propagation as transport of photon flux in a medium, from particle fluctuation to energy transport, to study transport of light energy in biological tissues problem. The theory pioneered by Arthur Schuster in 1903, first applied to the theory of gas dynamics and neutron transport. This method is mathematically lax due to ignoring the complex wave effects of photon transmission. However, due to the greatly simplified equations, the theory can flexibly handle the energy transfer phenomena in many random media [20] and is now widely used in many research fields [21–23].

1 $$ {\displaystyle \begin{array}{l}\frac{1}{c}\frac{\partial u}{\partial t}\left(r,\widehat{s},t\right)+\widehat{s}\cdot \nabla u\left(r,\widehat{s},t\right)+\left({\mu}_a+{\mu}_s\right)u\left(r,\widehat{s},t\right)\\ {}={\mu}_s{\int}_{4\pi }p\left({\widehat{s}}^{\prime },\widehat{s}\right)u\left(r,{\widehat{s}}^{\prime },t\right)\mathrm{d}{\Omega}^{\prime }+q\left(r,\widehat{s},t\right)\end{array}} $$

The above formula is the RTE time-domain expression. Where *r* denotes a certain point in the imaging space, Ω denotes the imaging space, *c* denotes the photon propagation rate in the biological tissue, *푡* denotes the time parameter, $u\left(r,\widehat{s},t\right)$denotes Radiance whose dimension is *Wm*^−2^sr^− 1^*Hz*^− 1^; *μ*_*a*_ and *μ*_*s*_ denote the absorption and scattering coefficients respectively representing the transmission of photons in the biological tissue. $p\left({\widehat{s}}^{\prime },\widehat{s}\right)$ denotes the normalized phase function representing no photonic phase, indicating that a single photon in a single scattering from the probability of scattering in the direction apparently satisfies the probability integral in the spatial domain of 1, ie. ${\int}_{4\pi }p\left({\widehat{s}}^{\prime },\widehat{s}\right)\mathrm{d}{\widehat{s}}^{\prime }=1$. $q\left(r,\widehat{s},t\right)$ denotes the spatial and angular distribution of the fluorescence to be sought [24–26].

The above radiation transfer equation is based on conservation of energy and is a very complex calculus equation. In three-dimensional biological tissue, the solution of RTE is transformed into a six-dimensional space-time problem. There are few methods in solving mathematical and computer problems, and it is usually not able to directly close the analytical solution. Moreover, because of its unknowns, it can be solved precisely only in rare cases. Usually it can not get a closed analytical solution. At the same time, it is extremely difficult to solve RTE directly, while the exact solution will only exist in rare cases. Therefore, it is common practice to replace itself with a simplified approximation of the radiation transfer equation [27].

Diffusion Equation (DE) is a widely used RTE-based simplified model [28–34]. It uses the first-order spherical harmonic function to expand the important function items in the RTE equation and performs the approximate processing, which significantly reduces the computational complexity and is suitable for the visible and near-infrared bands of the FMT imaging. The researches show that in the visible and near-infrared light bands, the result of DE and RTE has high similarity, so it becomes the mainstream model of optical imaging. In principle, DE is based on the approximation of RTE where the photon scattering coefficient is much greater than the absorption coefficient (μ_a_<<μ_s_΄). When photons do not meet these characteristics, such as the cavity inside a living organism, or highly scattering tissue like liver, it is difficult to obtain the exact solution of the DE equation. In addition, the diffusion equation takes the near-source error in describing the photon propagation near the light source region [7], which limits the application range of the diffusion equation.

Based on the above drawbacks of DE equations, in recent years, researchers have proposed such high-order approximations as RTE [7, 35–41]. Compared with diffusion equations, higher-order approximation models can significantly improve FMT accuracy. The SN model [42], the PN model [43], and the SPN model [41] are three commonly used RTE high-order approximation models and usually give more accurate RTE solutions to the more diffusive equations. By these approximation methods, the traditional RTE equation can be transformed into several coupled higher-order partial differential equations for easy calculation and solution. For example, two coupled equations of *N* (*N* + 2) and (*N* + 1) can be obtained using the SN model and the PN model, respectively. Here, N represents the number of Legendre Polynomials and direction cosines used for SN and PN approximation. As N increases, the approximate order increases. In recent years, SN models and PN models applied to optical molecular imaging have achieved good results [35, 44]. However, the RTE approximate solution based on the SN model and the PN model needs to solve multiple higher order partial differential equations at the same time, which causes great computational cost and limits its practical application. The SPN model is considered to be a good solution to the approximation of diffusion equations and to obtain the improved RTE solution. The SPN model only needs to solve (*N* + 1)/2 equations, much smaller than the SN model and the PN model, which improves its practical application. Taking *N* = 7 as an example, we only need to solve four coupled partial differential equations, the coupling equation is as follows [38]:

2 $$ {\displaystyle \begin{array}{l}-\nabla \cdot \frac{1}{3{\mu}_{a1}}\nabla {\varphi}_1+{\mu}_a{\varphi}_1=\\ {}S+\left(\frac{2}{3}{\mu}_a\right){\varphi}_2-\left(\frac{8}{15}{\mu}_a\right){\varphi}_3+\left(\frac{16}{35}{\mu}_a\right){\varphi}_4\end{array}} $$

3 $$ {\displaystyle \begin{array}{l}-\nabla \cdot \frac{1}{7{\mu}_{a3}}\nabla {\varphi}_2+\left(\frac{4}{9}{\mu}_a+\frac{5}{9}{\mu}_{a2}\right){\varphi}_2=\\ {}-\frac{2}{3}S+\left(\frac{2}{3}{\mu}_a\right){\varphi}_1+\left(\frac{16}{45}{\mu}_a+\frac{4}{9}{\mu}_{a2}\right){\varphi}_3-\\ {}\left(\frac{32}{105}{\mu}_a+\frac{8}{21}{\mu}_{a2}\right){\varphi}_4\end{array}} $$

4 $$ {\displaystyle \begin{array}{l}-\nabla \cdot \frac{1}{11{\mu}_{a5}}\nabla {\varphi}_3+\left(\frac{64}{225}{\mu}_a+\frac{16}{45}{\mu}_{a2}+\frac{9}{25}{\mu}_{a4}\right){\varphi}_3\\ {}=\frac{8}{15}S\hbox{-} \left(\frac{8}{15}{\mu}_a\right){\varphi}_1+\left(\frac{16}{45}{\mu}_a+\frac{4}{9}{\mu}_{a2}\right){\varphi}_2\\ {}+\left(\frac{128}{525}{\mu}_a+\frac{32}{105}{\mu}_{a2}+\frac{54}{175}{\mu}_{a4}\right){\varphi}_4\end{array}} $$

5 $$ {\displaystyle \begin{array}{l}-\nabla \cdot \frac{1}{15{\mu}_{a7}}\nabla {\varphi}_4+\Big(\frac{256}{1225}{\mu}_a+\frac{64}{245}{\mu}_{a2}+\\ {}\frac{324}{1225}{\mu}_{a4}+\frac{13}{49}{\mu}_{a6}\Big){\varphi}_4=\\ {}-\frac{16}{35}S+\left(\frac{16}{35}{\mu}_a\right){\varphi}_1-\left(\frac{32}{105}{\mu}_a+\frac{8}{21}{\mu}_{a2}\right){\varphi}_2\\ {}+\left(\frac{128}{525}{\mu}_a+\frac{32}{105}{\mu}_{a2}+\frac{54}{175}{\mu}_{a4}\right){\varphi}_3\end{array}} $$

In addition, in order to overcome the defect of the diffusion equation, the researchers also proposed some mixed photon propagation models that incorporate diffusion equations and other models. For example, The Radiosity-diffusion Model can be used to describe photon propagation in non-scattering regions [45]. However, the model is ineffective in low-scattering regions. In addition, Monte Carlo (MC) and the diffusion equation model (MC-DE) are also reported [46–48]. In the MC-DE model, the photon propagation near the light source is obtained by MC simulation, while in other imaging regions diffusion equations are used to describe this. Although MC describes photon transmission more accurately, its computational load is larger, which seriously affects the computational efficiency of MC-DE [49]. Accordingly, T. Tarvainen et al. proposed a mixed model combining RTE and DE equations [49]. The hybrid model uses RTE to describe photon transmission in the imaging region that does not satisfy the DE assumption, and the remaining regions are described using DE. The model can effectively characterize the photon propagation properties in high-scattering regions and correct the defects such as near-source error of DE. The results show that the hybrid model can approximate the same accuracy as RTE, and the computational efficiency can be effectively improved compared with RTE.

These reported photon propagation models have been widely used in FMT and achieved good results. However, computational efficiency and accuracy of photon propagation models require further study to significantly improve the accuracy and efficiency of FMT reconstruction. In addition, different photon propagation models need to be developed for different wavelengths and different imaging area sizes.

### Forward problem solving

The linear relationship between the measured data on the surface of the imaging area and the internal fluorescence distribution in the imaging area based on the photon propagation model is the core of the FMT forward problem. In recent years, researchers have proposed various mathematical solution methods including analytic method, statistical method and numerical analysis method to solve the forward problem of FMT [6, 7]. The analytical solution to RTE and its many approximation models is usually based on the Green’s function. The Green’s equation sets the light source to a δ-equation, and on the basis of which the light source is convolution expanded until it fills the entire imaging space [43]. Parsing solves quickly, but is limited to some special cases, such as homogeneous model contains simple and regular objects. Although analytic methods have been extended to imaging spaces of more complex geometries, such as multi-layered homogeneous plates [50], this method needs further study in FMT in complicated three-dimensional imaging space, especially in the imaging space containing complex geometry happening. Recently, the Kirchhoff Approximation (KA) has been utilized to solve the forward problem of FMT [51, 52]. Compared with the traditional analytical method, the KA has achieved relatively high computation efficiency, and high adaption to complex geometric models.

MC is a classical statistical method for solving photon propagation, which obtains photon propagation properties in imaging space by tracking the propagation of a large number of independent photons. Therefore, this method can be applied directly to solving RTE and is considered as the gold standard for solving forward problems [53]. MC is easy to implement and does not require excessive computational constraints, however, it requires relatively repetitive calculations, and reliable statistical results can only be obtained by computing large sample quantities of photons. Computing speed and memory footprint are the main factors that limit MC applications. In recent years, researchers have proposed a MC method based on GPU (Graphic Processing Unit) hardware acceleration [54–56], which significantly improves the computational speed compared with the single-CPU MC method. Meanwhile, the GPU-based MC simulation reconstruction algorithm has also been successfully applied to FMT reconstruction [55, 57].

Compared with analytic method and statistical method, numerical analysis method is the main solving method currently used in optical molecular imaging reconstruction. Its computational efficiency is high and its applicability is wide. Numerical analysis methods include Finite Difference Method (FDM) [40], Boundary Element Method (BEM), Finite Element Method (FEM) [37, 58] and Meshless Method (MM) method [59]. FDM uses equidistant grid points and regular grids to solve the forward problem, which is more efficient than irregular grids. However, FDM has difficulty in dealing with geometrically complex imaging spaces and boundary conditions. In contrast, FEM is the mainstream solution to FMT forward problems in recent years. The main advantage of FEM is its effectiveness in dealing with complex geometric problems. In addition, the system matrices obtained by FEM are usually sparse and positive definite, which leads to a more stable solution and high computational efficiency, which is also beneficial to FMT reconstruction [60, 61]. However, the main drawback of FEM is that it is difficult to generate FEM grid. In contrast, BEM only needs to discretize the imaging surface and the boundaries of the heterogeneous tissue within the space without the need to mesh the entire imaging space.

**Fig. 1:**
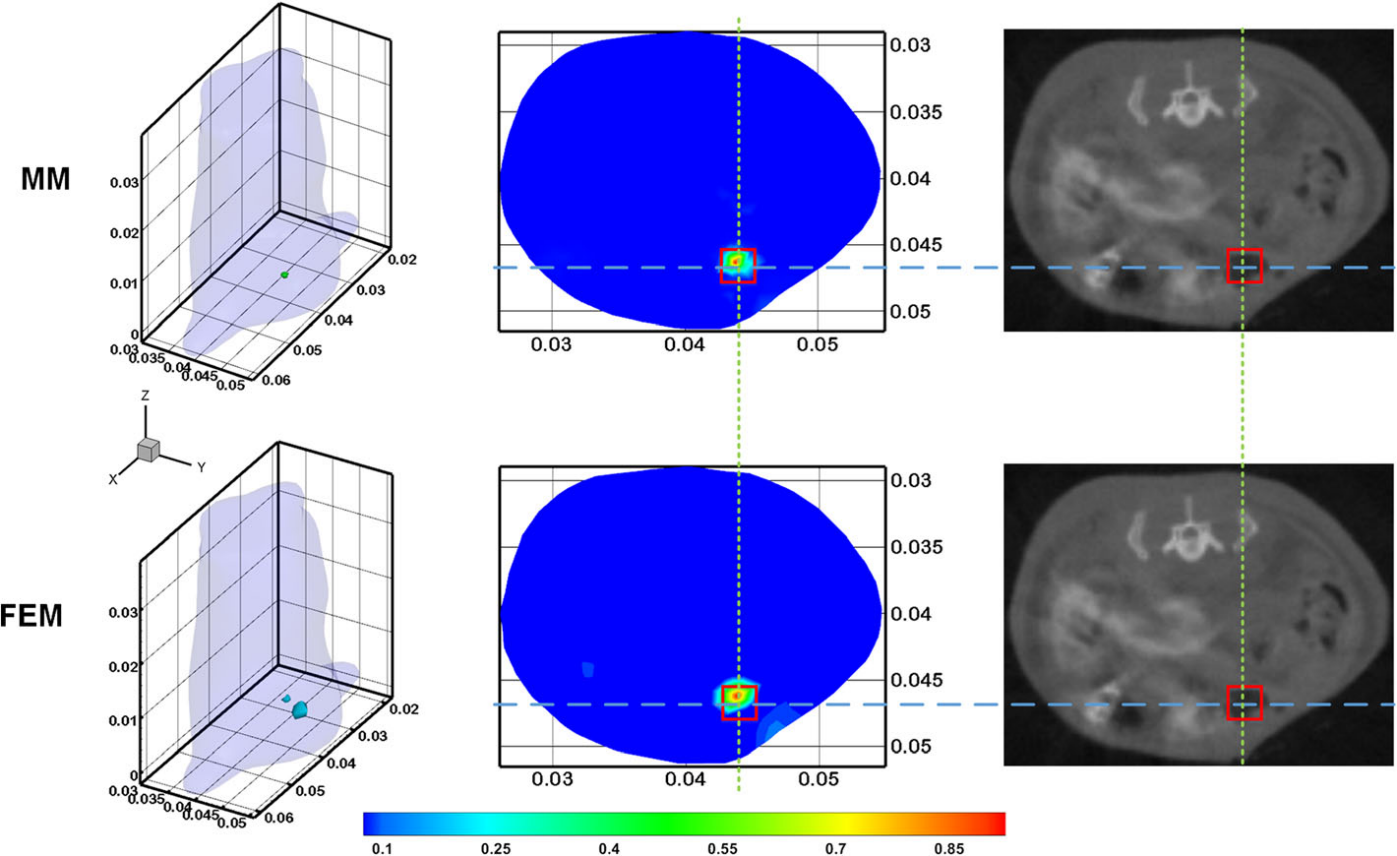
Reconstruction of the in vivo experiment based on meshless method (MM) and finite element method (FEM). The first row and second row list the results of MM and FEM, respectively. The first and second columns list the 3-D visualization and cross-sectional of the reconstructed fluorescent sources. The third column lists the corresponding micro-CT cross-sectional image. The red square markers clarify the actual locations of the fluorescent bead. The figure is reproduced from [62]

Therefore, compared with FEM, BEM can effectively reduce the computational dimension and complexity, to improve computational efficiency. However, fast and stable 3D mesh generation for complex geometry problems remains a challenging issue. In order to overcome the problem of 3D mesh generation, Y. An et al. proposed a meshless method [62] and applied it to solve the forward problem of FMT, and the result is shown in Fig. 1. The method only needs to obtain nodes that are relatively independent from each other to discretize the imaging space and does not require a cumbersome gridding process.

Regardless of which method is used to solve the photon propagation model, a linear relationship describing the three-dimensional fluorescence distribution of the surface measurement data within the imaging space during FMT imaging is finally obtained as shown in the following equation:

6 $$ AX=b $$

Among them, *A* ∈ **R**^*m* × *n*^ denotes the FMT system matrix, depicting the photon in the imaging space within the discrete nodes on the transmission characteristics. *m* is the number of surface measurement data, *n* is the number of internal nodes in the imaging space. *X* ∈ **R**^*n*^ represents the desired target value of each discrete node inside the imaging space. *b* ∈ **R**^*m*^ denotes the surface measurement data vector. Therefore, solving Eq. (6) is the core issue of FMT reconstruction, that is, the core problem of FMT inverse problem solving.

### Inverse problem solving

In FMT preclinical and clinical trials, *b* in eq. (6) is usually only measured from the imaging surface. However, the dimension of the measurement data on the imaging space surface is usually much less than the number of internal nodes in the imaging space, ie *m*>>*n*. Therefore, solving Eq. (6) is ill-conditioned [33]. Although the excitation fluorescence can be combined by multiple excitation-emission equations, the ill-condition of the inverse problem can be alleviated to a certain extent [63, 64]. However, the effective information contained in each excitation-emission system usually repeats each other and cannot be used as an effective solution information. Therefore, it cannot eliminate the ill-condition. Moreover, because of the high scattering properties of photons in the imaging space, Eq. 6 is ill-posedness and it is difficult to find the exact solution [65–67]. At the same time, the measurement noise generated during the experiment also affects the accuracy of the FMT reconstruction [68].

The ill-posedness of the FMT inverse problem is mainly due to the lack of information and uncertainty due to the high scattering of photons. In order to overcome the ill-posedness of reverse problems, researchers started from the light source prior information and combined with a variety of a priori information related to the light source and photon transmission to reduce the uncertainty of the information so as to improve the accuracy of inverse problem solving [15, 30, 69–78]. Feasible region is one of the earlier applied to the optical reconstruction of the information [79, 80]. The main idea is to infer the light source in the imaging area by the location of the light spot area generated by the surface measurement to approximate the area, so as to remove the nodes in other non-approximate regions in the imaging space, reduce the number of unknowns in Eq. (6) to handle the ill-posedness. However, the FMT imaging process is different from other imaging modalities due to the influence of the excitation light position. Usually, the spot generated by the surface of the living body has a strong correlation with the position of the excitation light. Therefore, it is determined directly from the surface spot that the feasible region of the internal light source exists the error. Therefore, feasible regions are not usually used for reconstructing during FMT reconstruction. Moreover, it is very difficult to use the feasible region effectively when the internal light source of the imaging region is in a deep position or there are multiple fluorescent light sources [81]. In order to improve these problems, researchers have proposed posterior feasible regions and optimized feasible regions [82, 83], and have been verified by numerical simulation models. The advantage of optimizing the feasible region is that it takes the whole imaging space as the initial feasible region, and optimizes the feasible region by iteratively updating the feasible region by judging the region where the light source is most likely to appear. On this basis, the researchers also proposed a feasible method of regional adaptive correction to further improve the applicability of feasible regions [84, 85].

The optical parameters (photon absorption coefficient and scattering coefficient) of each node in imaging space are the key factors that affect the reconstruction effect of FMT, and are the sufficient conditions that affect the photon propagation model and the accuracy of the reconstruction method [86–89]. The classical FMT method assumes imaging space as homogeneous space, that is, the optical parameters of each point in imaging space are the same. This assumption greatly simplifies the reconstruction operation, computational efficiency [90]. However, in biological conditions, the imaging space is usually not homogeneous, the optical parameters of various organs and tissues are far apart, and the reflection effects of different optical transmission media exist. Therefore, the researchers combined the prior information of the structure into the FMT reconstruction, proposed a non-homogeneous imaging space model and a priori reconstruction method, which greatly improved the reconstruction accuracy. The structure of imaging space prior information can usually be obtained by high-resolution structural imaging modalities such as computed tomography (CT), magnetic resonance imaging (MRI) [91–96]. The optical parameters of various organs and tissues can be obtained by other imaging techniques such as diffuse optical tomography (DOT) [97]. The imaging technique that combines imaging modalities to increase imaging prior information is also known as Multi-Modality imaging and is the focus of current medical imaging research [4].

Although researchers have put forward priori knowledge such as feasible regions, structural prior information to augment the information needed for reconstruction, the morbidity of the FMT reconstruction equation remains unresolved. Moreover, the actual FMT acquisition data usually contains a certain amount of noise, which has a great impact on the reconstruction of the pathological eq. A small signal disturbance may lead to a large reconstruction error. Therefore, researchers apply regularization techniques to FMT reconstruction to constrain the reconstruction process and reduce morbidity [8, 9, 28, 30, 63, 98–115]. The main principle of regularization is as follows:

7 $$ \Theta (X)=\Psi \left(X,b\right)+\lambda \nu (X) $$

where Ψ is the data fitting function, *v* is the regularization term, and *λ* is the regularization parameter, which is used to balance two items in Eq. (7). In FMT reconstruction, Ψ is usually chosen least squares, i.e. ${\left\Vert AX-b\right\Vert}_2^2$. Recently, researchers proposed a new data fit term that yielded better reconstructions [116]. The regularization term choice of eq. (7) is the key to reducing morbidity and suppressing noise. Lp-norm regularization is the commonly used regularization method for FMT reconstruction, which is ${\left\Vert X\right\Vert}_p^p$ [100, 114, 117]. The classical regularization term is *L*_2_ regularization, that is *p* = 2. The *L*_2_ norm regularization usually obtains a smoother reconstructed result of a large reconstructed area and has a good reconstruction effect for a large light source volume in an imaging space. Moreover, *L*_2_ norm can be derived mathematically, so it can be solved by many classical optimization methods, which is mathematically complete [118]. However, *L*_2_ norm reconstruction artifacts are usually large, and the phenomenon of smoothing occurs during the reconstruction, which is not conducive to the reconstruction of the complex structure of the light source.

**Fig. 2:**
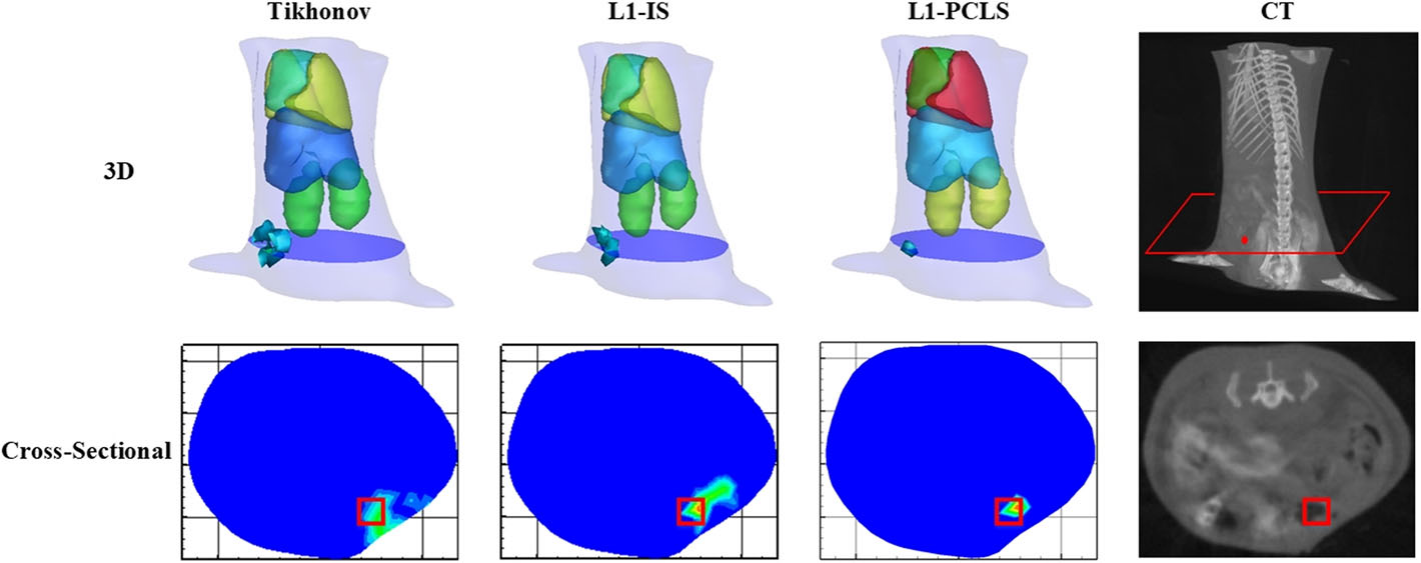
Views of the reconstruction results using *L*_2_-norm regularization (Tikhonov), *L*_1_-norm regularization (L1-Iteration Shrinkage, L1-IS) and *L*_1_-norm regularization piecewise constant Level-Set (L1-PCLS) methods. The blue plane in the figure is the z = 6.4 mm slice from the mice. The red dot marks the real position of the fluorescent bead. The figure is reproduced from [98]

Based on the regularization method, researchers began to try to apply the prior knowledge of light source to the regularization method. The priori knowledge that is currently used in regularization methods is the sparsity of fluorescent light sources. It is based on the assumption that the space occupied by a fluorescent light source is relatively sparse with respect to the whole imaging space or the distribution in the imaging space is sparse. In the actual biological tumor model, when the tumor is in the early stage of development, the tumor volume is relatively small; the distribution is more dispersed, more in line with the above assumptions. The regularization method based on sparsity is developed from the theory of compressed sensing (CS). Its basic principle is based on the signal sparsity characteristics, which recover the original signal from the missing collected information. *L*_1_ norm regularization (*p* = 1) is the mainstream sparse reconstruction method applied to FMT reconstruction today, which can reconstruct a good fluorescence three-dimensional distribution image based on less fluorescence acquisition information [10, 98, 108, 109, 113]. Numerical simulation, physical simulation and in vivo experiments verify that regularization of *L*_1_ norm and *L*_2_ norm regularization can achieve more accurate reconstruction results [113]. However, *L*_1_ norm regularization works well in reconstructing a sparse light source, but over-convergence also exists. Moreover, regularization of the *L*_1_ norm also makes it difficult to reconstruct good images when the fluorescent light source does not meet the sparsity characteristics. The comparison of the *L*_1_ norm and *L*_2_ norm regularization is shown in Fig. 2.

Besides the *L*_1_ norm and *L*_2_ norm regularization, other forms of Lp norm regularization were utilized in the reconstruction of FMT [100, 114, 117, 119, 120]. These regularization methods (0 ≤ *p* ≤ 1) not only make full use of the gradient information of the objective functions, like the Tikhonov method, but also retain the advantages of the sparsity regularizations in improving image quality.

**Fig. 3:**
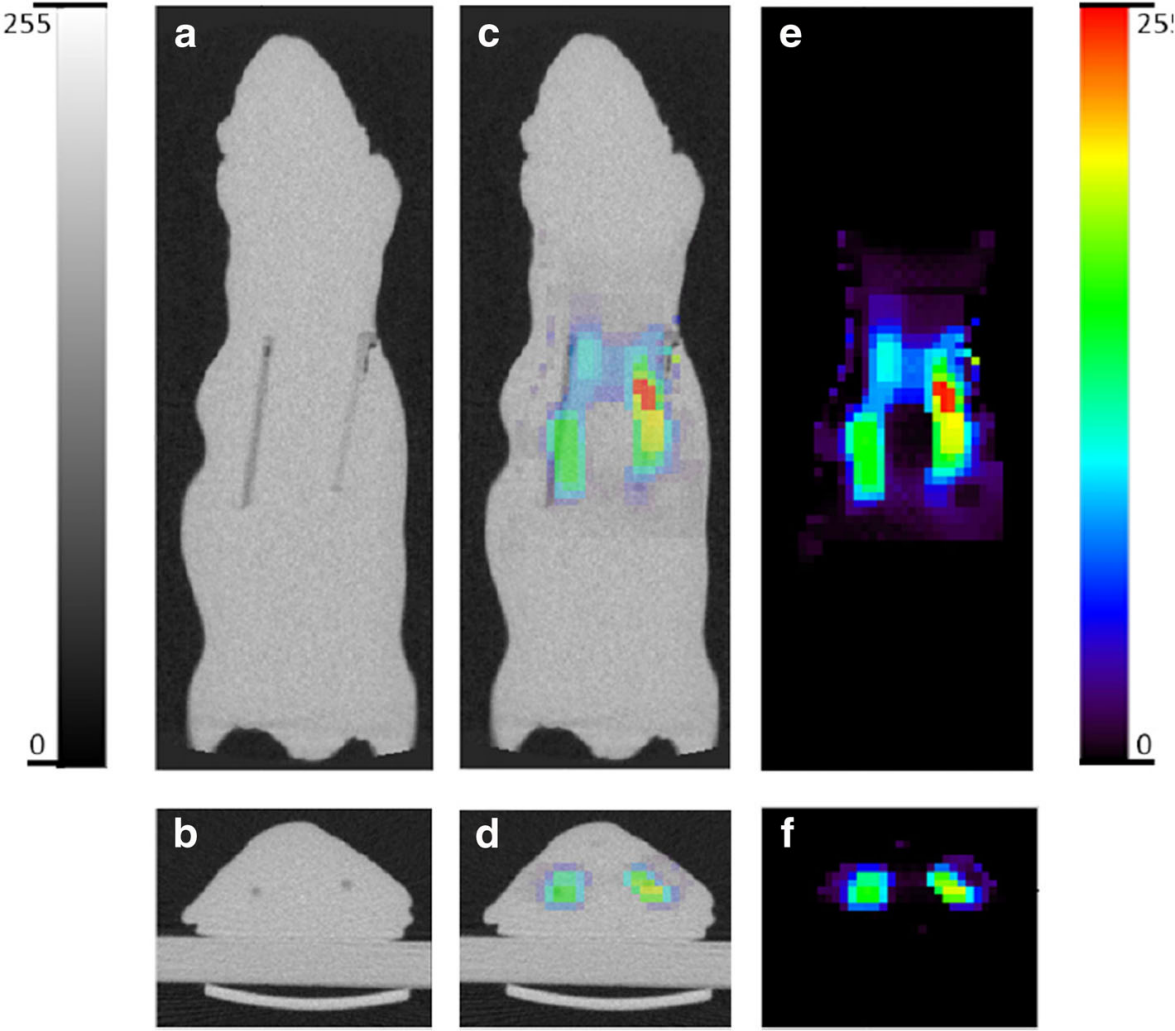
**a** Coronal and **b** transverse sections of the CT image of the mouse-shaped phantom showing the two embedded fluorescent line sources. **c** Coronal and **d** transverse overlay of CT and FMT images. **e** Coronal and **f** transverse sections of the FMT image showing the two fluorescent line sources reconstructed using both *L*1 and TV penalties with regularization parameters of 10 and 1, respectively. The figure is reproduced from [102]

Another available regularization method is total variation (TV) [102, 121]. TV norm regularization was first proposed by Rudin et al. and has been applied to image denoising, DOT, photoacoustic imaging and BLT in recent years [65, 122–125]. The main idea of TV norm regularization is to constrain the variation terms of the distribution of the fluorescent light sources while preserving the boundaries of the light source zones. However, the TV norm regularization implies the following assumptions: the region of the fluorescent light source and the surrounding non-fluorescent light source region have the characteristics of piecewise constant, that is, the light intensity difference between the light source region and the non-light source region is relatively large, and in each region light intensity is relatively constant [126–128]. In FMT reconstruction, FMT imaging satisfies the above assumptions when the property of the imaging internal light source is relatively simple (e.g., just one tumor). Therefore, the TV norm can be better applied to FMT reconstruction. The disadvantage of TV norm is its non-smoothness and non-differentiable, which makes it difficult to calculate. The traditional optimization method is difficult to apply TV norm solution. In order to solve this problem, the researchers put forward a variety of targeted optimization methods. Split-Bregman method is one of the representatives [129]. It decomposes the coupled complex optimization problems in TV norm regularization into two relatively independent sub-optimization problems, which can effectively improve the reconstruction effect. The experimental results show that the TV norm can get better reconstruction results. However, further analysis and improvement are needed. The comparison of *L*_1_ norm and TV norm regularization is shown in Fig. 3.

The reconstruction effect of regularization method is usually related to the choice of regularization parameter λ. In general, when the regularization parameter is small, many artefacts are generated due to noise amplification. When the regularization parameter is larger, the reconstructed image will have smoothed or over converged. To this end, researchers have proposed a series of methods for selecting appropriate regularization parameters such as L-curve, U-curve, cross-validation, discrepancy principle [130, 131]. However, Vogel pointed out that the choice of regularization parameters should be associated with a specific inverse problem. The above methods have been widely used in DOT and FMT reconstruction [132, 133].

Based on the regularization model of FMT, there is a large number of solving methods to solve the inverse problem, such as Bayesian-based method [95, 112, 134], iterated shrinkage [29, 72, 118], pursuit method [135, 136], Newton-based gradient descent method such as conjugate gradient method [109, 110, 137–139], Split-Bregman method [10, 129, 136, 140], etc. These optimization methods achieved good result in FMT reconstruction especially in the balance of solving accuracy and computation efficiency. However, in general, the accuracy, efficiency and robustness cannot get the optimization at the same time. Take Newton-based method as an example, it could achieve relatively robustness and accurate results of FMT, but the computation efficiency is unsatisfactory. Recently, in addition to proposing the new solving methods, lots of work concentrate on the improvement of these traditional optimization methods to improve the reconstruction performance, such as the utilization of structure priori [10, 141, 142], reweighted method [9, 108, 118], etc.

## Conclusion

In this paper, we proposed the recent methodology advances in FMT. We briefly introduced the photon propagation model for FMT based on the radiative transfer equation, and further elaborated the solution method of forward problem and inverse problem based on the photon propagation model. We summarized the current research progress in the methodology of FMT, and focused on improving the accuracy, speed, and robustness of FMT.

As an important part of molecular imaging technology, the research and application of FMT has made rapid progress in the past decades because of the wide variety of probes and strong imaging signals. The outstanding advantages of low experimental cost and non-invasive in vivo observation have been widely used in many preclinical and clinical studies in recent years. However, there still remain difficulties to be solved for FMT which are as follows:

1. More accurate and efficient photon propagation theory should be proposed. With the rapid development of computer hardware and software, especially with the support of large-scale parallel processing technology and its corresponding large-scale computer workstations, complex models (such as RTE) that could not be solved before are likely to become a reality today, and studies are more efficient. Higher-order calculus imaging model solution method is also one of the future research priorities.
2. Recently, studies on in vivo imaging are mainly based on the use of specific probes to target in vivo tumors. In this regard, the excitation FMT technique in these researches is still in a preliminary stage and has not been widely implemented. Different from the implanted light source and simulation model, it is difficult for non-invasive real-life tumors to obtain information on the lesion inside the organism. Invasive imaging methods, such as frozen section imaging, have a certain degree of deformation in the same body condition. Therefore, how to verify the accuracy of reconstruction is also a problem that needs further study.
3. The goal of FMT is to obtain structural distribution information at the cellular and molecular level targeted to the lesion area, strongly dependent on photon propagation in biological tissues, and the physiological and pathological information that can be acquired by FMT has limitations. Integrating optics, structure, and functional imaging with each other, and multi-angle, systematic and comprehensive acquisition of multimodal fusion imaging has become the current research trend in the field of molecular imaging. At present, the more mature fusion-excited fluorescence tomography is combined with structural/optical multimode imaging represented by CT and MRI, functional/optical multimode imaging represented by PET, and optical/structure/functional multimodality image fusion. Both have received extensive attention and have published many high-level results in preclinical and clinical applications and imaging theory. In the future work, the methodology in multimode fusion imaging need to be studied, to extend the existing imaging theory and methods to multimode fusion imaging, and further improve the application of FMT and imaging quality.
